# Odor Experiences during Preimaginal Stages Cause Behavioral and Neural Plasticity in Adult Honeybees

**DOI:** 10.3389/fnbeh.2016.00105

**Published:** 2016-06-03

**Authors:** Gabriela Ramírez, Carol Fagundez, Juan P. Grosso, Pablo Argibay, Andrés Arenas, Walter M. Farina

**Affiliations:** ^1^Laboratorio de Insectos Sociales, IFIBYNE-CONICET, Departamento de Biodiversidad y Biología Experimental, Facultad de Ciencias Exactas y Naturales, Universidad de Buenos Aires, Pabellón II, Ciudad UniversitariaBuenos Aires, Argentina; ^2^Instituto de Ciencias Básicas y Medicina Experimental, Instituto Universitario del Hospital ItalianoBuenos Aires, Argentina

**Keywords:** preimaginal odor experiences, behavior, neurobiology, electrophysiology, honeybee

## Abstract

In eusocial insects, experiences acquired during the development have long-term consequences on mature behavior. In the honeybee that suffers profound changes associated with metamorphosis, the effect of odor experiences at larval instars on the subsequent physiological and behavioral response is still unclear. To address the impact of preimaginal experiences on the adult honeybee, colonies containing larvae were fed scented food. The effect of the preimaginal experiences with the food odor was assessed in learning performance, memory retention and generalization in 3–5- and 17–19 day-old bees, in the regulation of their expression of synaptic-related genes and in the perception and morphology of their antennae. Three-five day old bees that experienced 1-hexanol (1-HEX) as food scent responded more to the presentation of the odor during the 1-HEX conditioning than control bees (i.e., bees reared in colonies fed unscented food). Higher levels of proboscis extension response (PER) to 1-HEX in this group also extended to HEXA, the most perceptually similar odor to the experienced one that we tested. These results were not observed for the group tested at older ages. In the brain of young adults, larval experiences triggered similar levels of *neurexins (NRXs)* and *neuroligins (Nlgs)* expression, two proteins that have been involved in synaptic formation after associative learning. At the sensory periphery, the experience did not alter the number of the olfactory sensilla placoidea, but did reduce the electrical response of the antennae to the experienced and novel odor. Our study provides a new insight into the effects of preimaginal experiences in the honeybee and the mechanisms underlying olfactory plasticity at larval stage of holometabolous insects.

## Introduction

The olfactory system decodes the intricate matrix of chemical stimuli of the environment, extracting crucial information that enables animals to make decisions in diverse behavioral contexts. Odor detection and processing capacities of the olfactory system can change after chemosensory experiences. This great plasticity is remarkable early in life, when neural circuits are maturing and the system remains very sensitive to external stimuli (Masson et al., 1993; Knudsen, 2004). The honeybee is considered a model species within the invertebrates to study the behavioral and neural plasticity caused by early experiences (Masson and Arnold, 1984, 1987; Winnington et al., 1996; Sigg et al., 1997; Farris et al., 2001; Brown et al., 2004). Considering that some brain progenitor cells present at the larval stages are retained through metamorphosis (Farris et al., 1999; Consoulas et al., 2000), to what extent larval experience determines changes in the nervous system and behavior of the adult honeybee is less well known.

With a holometabolous development that includes larval, pupal and adult stages, the honeybee undergoes a complete metamorphosis by which not only the structure of its body, but also the organization of the nervous system changes. Holometabolous insects present two independent events of neurogenesis: the first during the embryonic development, which gives rise to the larval neurons; and the second during larval life and early metamorphosis, which provides adult-specific neurons. Some neuropils such as the adult ALs start the development in the late pupa but extent to the first days of adult life (Devaud et al., 2003). Most, but not all, sensory neurons are replaced by newly born neurons from imaginal disks (Tissot and Stocker, 2000). On the contrary within the central nervous system (CNS), many larval neurons are fated to die during metamorphosis. However, a large proportion of adult neurons derives from functional larval neurons that undergo changes in dendritic morphology, functional properties and synaptic interactions (Consoulas et al., 2000; Tissot and Stocker, 2000). Most larval motoneurons, some interneurons and several modulatory neurons persist to participate in significantly different behavioral patterns of the adult (Tissot and Stocker, 2000; Gerber and Stocker, 2007). Together with the remodeling of the nervous system, the extremely different habitats and behavioral repertoires of holometabolous insects before and after the adult emergence challenge the persistence of larval circuits through metamorphosis as well as the abilities to retrieve early experiences irrespective of the context. Several studies using different insect species with true metamorphosis provide evidence that larval experience can indeed influence adult behaviors (beetle: Alloway, 1972; ants: Isingrini et al., 1985; Carlin and Schwartz, 1989; moths: Rojas and Wyatt, 1999; Blackiston et al., 2008; Shikano and Isman, 2009; fly: Tully et al., 1994; Ray, 1999; solitary bee: Dobson, 1987; parasitic wasp: Gandolfi et al., 2003).

As far as we know only few studies addressed the role of preimaginal experiences in honeybees. By means of a passive olfactory exposure during the pupal stage, Sandoz et al. (2000) could not detect any effect neither in the orientation nor in the appetitive response of exposed bees. This result suggests that pupae were not sensitive to acquire the odor information that diffused through the wax cap. Under less well controlled experimental conditions, Boelter and Wilson (1984) tested the influence of pollen species ingested during the larval and young adult stages of the honeybee. The authors failed to condition foragers to a specific pollen type after their colonies had been fed the resource in question for 6 weeks (that is, when tested foragers still underwent the larval stage). Despite the mentioned evidence, we believe more experiments are required to bring light into the effect of preimaginal experience on adult honeybees.

New evidence relates the regulation of synaptic connectivity following sensory experience in adult honeybees (Reinhard and Claudianos, 2012). The adhesive synaptic molecules, the presynaptic *neurexins* (*NRXs*) and their postsynaptic binding partners, the *neuroligins* (*Nlg*s) are two highly conserved proteins of the synaptic membranes of neurons, which together form a trans-synaptic bond that assists synapse formation (Craig and Kang, 2007). Expression of both molecules was found in honeybee brain tissue with expression present throughout development and significantly up-regulated in adults (Dean and Dresbach, 2006). Interestingly, expression of both molecules was increased in the brain of foraging age bees after being successfully conditioned to an odor (Biswas et al., 2010), opening up the possibility to use their expression to search for larval memory traces in the adult honeybee.

An alternative mechanism by which larval experiences may persist through metamorphosis includes changes in the peripheral nervous system (PNS) after early stimulation. It is known that the conversion from the larval to adult sensory system during the late larval and early pupal stages involves the degeneration of all larval sensory organs and most of the associated sensory neurons, followed by the born of new sensory neurons and organs derived from sensory mother cells singled out in the imaginal discs (Meinertzhagen, 1989; Taylor, 1989; Hildebrand et al., 1997). Still, some larval sensory neurons survive through the pupal or even the adult molt (Tix et al., 1989; Lakes-Harlan et al., 1991; Shepherd and Smith, 1996; Consoulas et al., 2000) and may play a role, if not sensing the environment, in the assembly of the adult sensory system, for example by guiding axons during the establishment of the new sensory pathways of the adults. In hemimetabolous insects, several studies reported that feeding artificial diet produced a reduction in the number of antennal chemosensilla compared with plant-fed individuals (Rogers and Simpson, 1997). The variation in the sensilla number was also found after adding odors to the food (Bernays and Chapman, 1998), suggesting that sensory stimulation can modulate the number of these sensory organs. Although variation in the number of antennal sensilla was not reported in honeybee according to early experiences, there is evidence that both olfactory deprivation and experiences with odors induced modifications in the neural response of the antenna. Masson and Arnold (1984) observed that olfactory deprivation since emergence of the adults resulted in a decreased antennal response to different novel odors. Pioneer studies showed that electroantennogram (EAG) recordings of the whole antenna were increased with odor learning (De Jong and Pham-Delègue, 1991; Wadhams et al., 1994). Others detected no effect to the conditioning odors (Bhagavan and Smith, 1997; Sandoz et al., 2001). More recently, a reduction in the EAG response of odor conditioned honeybees was reported. Such a reduction correlated with changes in the expression of olfactory receptors (Claudianos et al., 2014). So far the contribution of morphological and functional changes in the antenna to the olfactory plasticity across metamorphosis remains unknown.

Feeding scented food as early as few days after emergence have been reported to modify behavioral responses of honeybees at foraging ages (Arenas and Farina, 2008; Arenas et al., 2012) and also the function and the structure of their antennal lobe (Arenas et al., 2009, 2012) that might reflect the reorganization of the brain connectivity underlying the formation of long-term memories. The food distribution inside the beehives also includes larvae as receivers (Crailsheim, 1998). Then odors from the larval food may also be learned and affect later behaviors. With this in mind, the aim of our study was to investigate how olfactory cues experienced at the larval stage impacted the later sensory and cognitive abilities of the honeybee. To provide individuals with a preimaginal odor experience, we fed colonies that homed larvae scented sugar solution. Under this scenario, we provided a controlled stimulus in the rearing environment of the worker-larvae that may lead to the formation of odor memories within an appetitive context.

Due to the fact that honeybees extend their proboscises as a reflex response to antennal stimulation with sugar (Frings, 1944) and because this response can be conditioned to an odor (Takeda, 1961), we used the proboscis extension response (PER) to conditioned bees of 3–5 and 17–19 days of age that had undergone a preimaginal experience. At these particular ages we compared the 1-hexanol (1-HEX) conditioning performance of bees that had been reared in colonies fed 1-HEX vs. the performance of bees that had been reared under the same conditions but in colonies fed unscented food (controls). Because early experiences with an odorant in the appetitive context of adult bees improved the retrieval and acquisition of the experienced odors (Arenas and Farina, 2008), we predict that preimaginal exposure to 1-HEX scented food would also induce high response levels to the known odor.

To evaluate memory retention to 1-HEX after metamorphosis and to investigate whether olfactory experience elicits a specific response (i.e., only affected the odor used for the stimulation or if the experience extents to other odors), we quantified PER to the stimulation odor 1-HEX and to three other novel odors that presented different degrees of chemical similarity with 1-HEX. To further search for a larval memory trace, we assessed the regulation of *Nlgs* (four alternatively spliced transcripts) and *neuroxines* in the brain of young adult honeybee with or without the controlled larval experience. Expression levels of these proteins were estimated by quantitative real time PCR amplification. Finally, to test odor detection after the larval olfactory experience we carried out EAG recordings in the antennae of 3–5 day-old bees. In an attempt to correlate the number of sensilla placoidea in the antenna with changes in its neural response, we reconstructed the three dimensional surface of the antenna and investigated the morphological changes that might have been triggered by the larval odor experience.

## Materials and Methods

### Study Site, Colonies and Caged-Bees

The experiments were performed during three consecutive seasons between October and April at the experimental field of the University of Buenos Aires, Argentina (34° 32′ S, 58° 26′ W). We used 10-frame hives of European honeybees *Apis mellifera* located in the experimental apiary. Several hives were used as *donor colonies*, untreated colony from which we selected and took brood frames. These frames, mainly containing newly laid eggs, were located into the *stimulation colonies* that were fed unscented or scented food in order to provide an odor in the rearing environment of the incoming larvae. We used a total of 12 colonies (e.g., two colonies fed unscented food and two colonies fed scented food per each experimental season).

Frames with the eggs that hatched into the experimental colonies, were marked and kept in the *stimulation colonies* until their brood cells were all capped. Once the brood combs were completely sealed, the frames were placed in an incubator under controlled conditions (36 °C, 55% RH and darkness). During the following days, the adults emerged. The recently emerged adults (0–1 days of age) were collected in groups of about 120 individuals into wooden cages (10 × 10 × 10 cm) with a single screen in one side. Laboratory cages offered unscented 50% weight/weight (w/w) sucrose solution placed in plastic tubes (10 mL volume) with a small opening (1 mm diameter) at the tip (for details, see Arenas and Farina, 2008). In addition, a second feeder containing pollen *ad libitum* was offered. Caged bees were kept in an incubator (30 °C, 55% RH and darkness) until the bees were used in different experiments.

### Odor Experience During the Larval Stage

To provide individuals with an olfactory experience at their larval stage, we fed two of the *stimulation colonies* sugar solution (50% w/w sucrose solutions) scented with 1-HEX from Sigma Aldrich; 100 μl of the pure odor per liter of solution). The other two *stimulation colonies* were fed unscented solution. Larvae reared in stimulation colonies fed unscented solution were used as controls. Scented and unscented foods were poured in hive feeders (1.5 l) to generate the appropriate environments for larval stimulation (see “Supplementary Material” for additional assay).

By dyeing the artificial food with neutral red (Red Amaranth, Saporiti^®^), we confirmed that colonies stored the artificial solution into the reserves of the nest (see “Supplementary Material”). Moreover, we could observe that nurse bees used the added food to feed the larvae, as the jelly that the nurses supplied inside the cells was stained light red. With evidence that the solution we offered was available in the brood cells, we used the offering of scented food to give the larvae an experience with an odor.

### Testing Behavioral Responses

In order to evaluate the effect of preimaginal olfactory experiences on adult response, experimental bees were tested in the PER paradigm (Takeda, 1961). Due to the fact that honeybees can retrieve early olfactory experiences acquired in an appetitive context by the extension of the proboscis (Takeda, 1961), we reasoned that odor cues experienced in the food of the larvae could also be recovered in the PER paradigm.

Bees reared in *stimulation colonies* fed unscented food were also tested. Experimental bees were evaluated at the age of 3–5 and 17–19 days. Bees were taken from their cage at the chosen age, anesthetized by chilling at −4 °C and harnessed in small metal tubes that just allowed the movement of antennae and mouthparts. Only bees that extended their proboscises after applying 50% w/w sucrose solution to the antennae (i.e., bees that showed the unconditioned response) and did not respond to the mechanical air flow that would release the odor, were tested. For the odor stimulation, a constant and clean air stream (2.5 ml/s) was directed to the antenna of the bee by means of a device connected to a computer. Controlled by the computer, the air stream was redirected to pass through a syringe with a filter paper (30 × 3 mm) imbibed in 4 μl of 1-HEX for the odor presentation. The whole trial lasted for 46 s and consisted of 20 s of clean air, 6 s of odor, and 20 s of clean air again.

#### PER Conditioning to 1-HEX

To test whether bees that experienced 1-HEX during their larval stage show different learning abilities than control bees, adult bees were conditioned to 1-HEX in the PER setup. Olfactory PER conditioning consisted in pairing the presentation of 1-HEX conditioned stimulus (CS) with a drop of 50% w/w sucrose solution as a reward unconditioned stimulus (US) to establish an association between the stimuli. The whole trial lasted for 46 s and consisted of 20 s of clean air, 6 s of odor, and 20 s of clean air again. In each trial, the US was presented during the last 3 s of the odor presentation by stimulating the antennae and offering the reward on the proboscis. The inter-trial interval was 15 min. This olfactory conditioning consisted in four consecutive trials. A conditioned response (CR) was considered positive if the bee protruded its proboscis during the first 3 s of CS presentation with no need of touching the antennae with the reward.

#### PER to 1-HEX and to Novel Odors

To test how bees responded to 1-HEX (the odor they had experienced at the preimaginal stage) and to other three novel odors that present different degrees of chemical similarity with it, we measured PER to the first presentation to 1-HEX, 1-Nonanol (1-NON); Nonanal (NONA) and Hexanal (HEXA). Novel odors differed in two dimensions with the primary alcohol of six carbons, 1-HEX: the carbon-chain length and functional group. Namely HEXA is an aliphatic aldehyde of six carbons, 1-NON is a primary alcohol of nine carbons and NONA is an aliphatic aldehyde of nine carbons.

The presentation of the odors was carried out following the same procedure we used in previous experiments. Each odor was presented only once, in a random sequence and with a 15 min inter-trial between the successive presentations. After the presentation of all the four odors, PER to sugar was tested to check bees capacity to respond. Bees that did not show the proboscis extension reflex to the reward were discarded from the analysis.

### Brain Dissection and RNA Extraction

Brain tissues were obtained from 3–6 day-old honeybees with or without preimaginal olfactory experiences. Bees were placed in a freezer under −200 °C for approximately 3 min. The head of each bee was removed using a scalpel and fixed with bee wax. We covered up the head with physiological solution while the frontal section of the head capsule was carefully removed to reveal the brain. The glands were removed using forceps. Finally, the brain was gently pulled out of the head and put it on a cryovial. Ten brains from the same experimental group were located per cryovial and stored in liquid nitrogen (under −200 °C approximately) until ribonucleic acid (RNA) extraction.

Total RNA was isolated using Trizol reagent (Invitrogen Life Technologies^®^, MA, USA). Adult brain RNA extraction was conserved under −70 °C. An aliquot of RNA was then used for gel electrophoresis to assess the integrity of the extraction using 1% agarose gel. RNA samples were then quantified by spectrophotometry since a 1/100 dilution (1 μl de RNA in 99 μl of filtered water). To avoid any deoxyribonucleic acid (DNA) contamination 4–5 μg of RNA were treated with “RQ1 *DNAsa*” enzyme (Promega^®^, WI, USA). After this treatment the RNA was quantified by spectrophotometry and 1 μg was used to synthesize cDNA. Using cDNA as model reverse transcription was controlled by means of quantitative Real Time PCR (qRTPCR). qRTPCR reactions were made in a Lightcycler 2.0 Instrument (Roche^®^) using Taq Platinum (Invitrogen^®^) enzyme and SYBER Green I (Invitrogen^®^) dyeing. The qRTPCR primer sequences for the constitutive gen RPL8 as well as for the *Nlg2–5* and *Nrx1* were taken from Biswas et al. (2008). All primer sequences, the size of amplification product and the annealing are describe in Table 1. To test the specificity of the primers for *Apis mellifera* as well as the sequence hybridization we aligned the sequences using BLAST tool. Primers were optimized with cDNA from a bee brain pool. The presence of a unique product of expected molecular weight was verified in a 3% agarose gel. The reaction efficiency for each primer was calculated by mean of a calibration curve with dilutions from the cDNA samples (1/20, 1/40, 1/80 and 1/160). We employed 10 μl of template from a 1/40 dilution. Each sample was amplified trice. We normalized the obtained value for each gene to the constitutive *RPL8* gene whose expression does not change between tissues or between the stages of the bees (Collins et al., 2004). Normalized gene expression values were compared between adult honeybee brains with different preimaginal olfactory experiences.

**Table 1:** **Primers models, amplified size product and annealing for the constitutive gene (*RPL8*) as well as for Neuroxin 1 (*Nrx1*) and Neuroligins 2–5 (*Nlg 2, Nlg 3, Nlg 4* y *Nlg 5*)**.

Primer	Sequence (5′ to 3′)	Product size (MW)	Annealing
*RPL8*	F: CACACGGTGGTGGTAATCAT	114pb	56
	R: CTCGGATTCTTCCTGTACGA
*Nlg2*	F: GGTGTTCCTCCTCGTGCTCAA	68pb	59
	R: ACGAGTTCCTGTCCCTCTGGTA
*Nlg3*	F: CATAGAGCTCAAGTCGAAACTGAA	124pb	56
	R: GAGAAGATGATGCGATCTAGGAA
*Nlg4*	F: CTTCCTGATTCTCGTCTGTCTGA	71pb	56
	R: GTGGATTCAGCTTGCTCTTGA
*Nlg5*	F: GGTTGTATTCTGTTGGTGCTCAATA	67pb	55
	R: TGTCTCGATCCCTCTGATAGTAAA
*Nrx1*	F: TCGAGTTCAAGACCGAGCA	81pb	57
	R: GCTTCGCCTCGAAGAAGTC

*These models were made up using the Primer 3 software. Their thermodynamics properties were evaluated with the Invitrogen Vector NTI AdvanceTM 10 software*.

### Electroantennogram (EAG) Recordings

The aim of this experiment was to test the effect of preimaginal stimulation on the electrophysiological response of the whole antenna. The antennae were taken from bees of 3–5 days of age that had been reared in *stimulation colonies* either fed 1-HEX-scented or unscented food. For the preparation, the antennae were cut at the scape using a micro-scissors under the stereo-microscope. The tip of the most distal segment of the flagellum was cut using a fine dissecting knife. Both the base and the top of the antenna were inserted into a drop of a conductive gel (SPECTRA 360 GEL) placed on the metal extremes of the holder (Syntech). These extremes act as the reference and register electrodes and enable the electrical current to circulate through the antenna placed into the holder. The holder was connected to a preamplifier (Syntech) and both were connected to a custom made amplifier. The recordings were performed using analogical-digital converter (PicoTest) and a custom made software. Before the stimulation, a baseline was obtained around 0 mV. Each antenna was recorded throughout an assay through which six different stimulations were performed. We used three different concentrations (1/100; 1/10 and 1/1) of two odors (1-HEX and NONA) to stimulate the antennae. We first presented 1-HEX (the odor which bees had experienced at the preimaginal stage) and then the novel odor, NONA. The concentrations were presented in an increasing manner. Unlike the previous experiments, odors were presented for just 1 s. Before the odors presentation, the response of air stimulation was recorded to know the contribution the mechanical stimulation made to the whole EAG response. For each antenna we recorded one EAG peak per stimulation. We quantified the maximum deflection obtained during the stimulation.

### Sensilla Counting

The aim of this experiment was to determine whether the morphology of the adult bee-antenna was modified after a preimaginal olfactory experience. We quantified the number of olfactory sensilla (e.g., placoidea) in different segments of the flagellum (7th and 8th). Antennae of 3–5 day-old bees with or without preimaginal olfactory experiences were dissected. Firstly we removed the antennae from 3–5 day bees at the pedicel using a scalpel under the stereo-microscope. Secondly, we placed the antenna on a slide with a drop of liquid glycerin to maintain the humidity of the preparations and we covered it with a slide-cover. The preparations were observed under the confocal scanning microscope (Olympus FV1000) that exited the preparation with a 480 nm laser. The auto-florescence of the cuticle allowed us to observe the antennal morphology. Using this microscope we reconstructed three-dimensionally 75% of the antenna surface from the 7th and 8th segments of the flagellum. The quantification of sensilla placoidea per segment was made using ImageJ^®^. We counted the number of sensilla placoidea inside a particular measuring area which was equivalent between antennae (see “Results” Section for details). We compared the number of sensilla placoidea between antennal segments as well as between experimental groups.

### Statistical Analyses

PER conditioning between bees reared in colonies fed 1-HEX-scented and unscented food was compared by applying a Generalized Linear Mixed Model (GLMM, Baayen et al., 2008) with binomial error structure and logit link function, including treatment as a fixed factor and bee as a random one.

PER responses to the stimulation and novel odors were compared using an analysis of variance for repeated measure, RM-ANOVA. Monte Carlo studies have shown that is possible to use ANOVA on dichotomous data (Lunney, 1970). When statistical differences in principal factors were found, Dunnet *post hoc* comparisons were carried out. Then if statistical differences were detected in the interaction between factors, simple effects were computed using the corresponding error and afterwards performed Tukey comparisons between factors (Sokal and Rohlf, 2000).

The effects of larval rearing environment on gene expression were analyzed performing a MANOVA using Infostat Software, threshold cycle number was relativized to Rpl8 expression and corrected for differences in primer efficiency.

To analyze the amplitude of EAG peaks, we applied a global analysis using a Generalized Linear Model (GLM) with negative-binomial error structure and logit link function. For this analysis, the fixed effect was the treatment and the dependent variable was the voltage (e.g., EAG recordings). GLM and GLMM models were fitted in R 3.2.1 (R Development Core Team, 2011) using the function glmof the R-package MASS (Venables and Ripley, 2002) for the GLM and the function lmer of the R-package lme4 (Maechler and Bates, 2010) for the later.

To compare if the morphology of the adult bee-antenna was modify after preimaginal olfactory experiences we used a two-factor analysis of variance (two-way ANOVA). One factor was the preimaginal olfactory experiences and the other was the number of the antennal segment (Sokal and Rohlf, 2000).

## Results

### The Effect of Preimaginal Experience on Later Behavioral Responses

**Figure 1:**
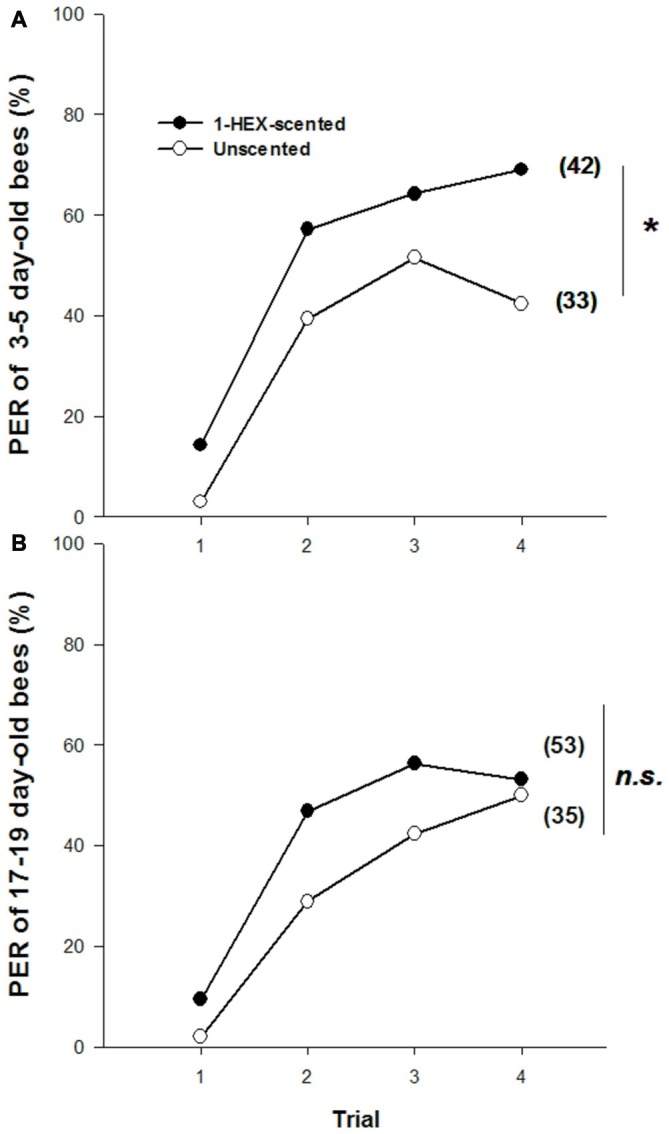
**Olfactory proboscis extension response (PER) conditioning to 1-hexanol (1-HEX) in adults that underwent a controlled olfactory experience at preimaginal stages.** Percentage of PER to 1-HEX through a 4-trial conditioning, in 3–5 **(A)** and 17–19 day-old bees **(B)** reared in colonies fed either 1-HEX-scented food (filled circles) or unscented food (empty circles) at their larval stage. Significant differences in PER values compared to control are labeled with *Pr(>|z|) < 0.05 (**A**,**B**, Generalized Linear Mixed Model (GLMM) test). The number of observations is shown between brackets.

Olfactory PER conditioning to 1-HEX (i.e., the experienced odor) differed between young adults that had been reared in colonies fed either 1-HEX-scented or unscented food. Three to five day-old bees reared in colonies fed scented food responded more throughout the conditioning than the control group (1-HEX-scented vs. Unscented colonies; GLMM: Pr(>|z|) = 0.03091; Figure 1A). However, no differences were detected in 17–19 day-old bees (GLMM: Pr(>|z|) = 0.383; Figure 1B).

**Figure 2:**
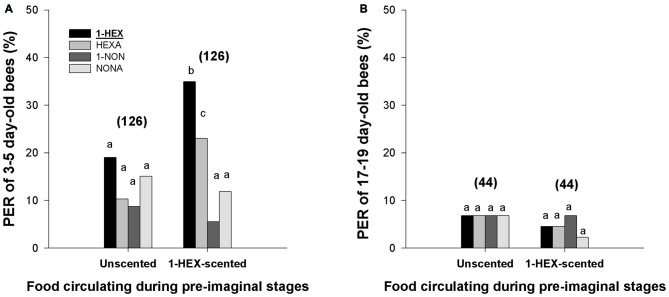
**Response to the stimulation odor and to novel odors in adults that underwent a controlled olfactory experience at preimaginal stages.** PER of 3–5 day-old bees **(A)** and 17–19 day-old bees **(B)** to the odor experienced during their preimaginal stages (1-HEX) and to three novel odors (HEXA, 1-NON and NONA). Individuals were reared in colonies fed either 1-HEX-scented or unscented food at their larval stage. Different letters indicate significant differences (two-way RM-ANOVA). The number of tested bees is shown in brackets.

Regarding the PER to the stimulation odor and to the novel odors, the analysis revealed that experienced bees of 3–5 days of age responded differently to the tested odors (F_(3,1000)_ = 16.419, *p* < 0.0001; Figure 2A). *Post hoc* comparisons showed that the bees presented higher PER response to 1-HEX and HEXA (e.g., the novel odor with a carbon chain of the same length) than to 1-NON and NONA (*p* < 0.05, Tukey comparisons, Figure 2A). Furthermore, higher PER response to 1-HEX was observed when compared with HEXA responses (*p* < 0.05, Tukey comparisons, Figure 2A).

Differences were also found between 3–5 day-old bees fed unscented and bees fed 1-HEX-scented food (two-way RM-ANOVA: F_(3,750)_ = 6.699, *p* = 0.00018; Figure 2A). Simple-effect analysis showed that differences between the groups were explained by differences in the PER levels to 1-HEX (F_(1,1000)_ = 12.396, *p* = 0.00045; Figure 2A) and HEXA (F_(1,1000)_ = 7.933, *p* = 0.00494; Figure 2A).

On the contrary, no difference in PER to all the four odors was found among 17–19 day-old bees. Two-way RM-ANOVA revealed no statistical differences among tested odors (F_(3,536)_ = 0.246, *p* = 0.864; Figure 2B) or between bees that experienced the 1-HEX diluted in their food and bees that were reared in colonies fed unscented food (F_(3,536)_ = 0.361, *p* = 0.549; Figure 2B). These results supported the survival of larval memories until young adult ages but not until foraging ages.

### The Effect of Preimaginal Experience on the Regulation of Neuroligins and Neurexin

**Figure 3:**
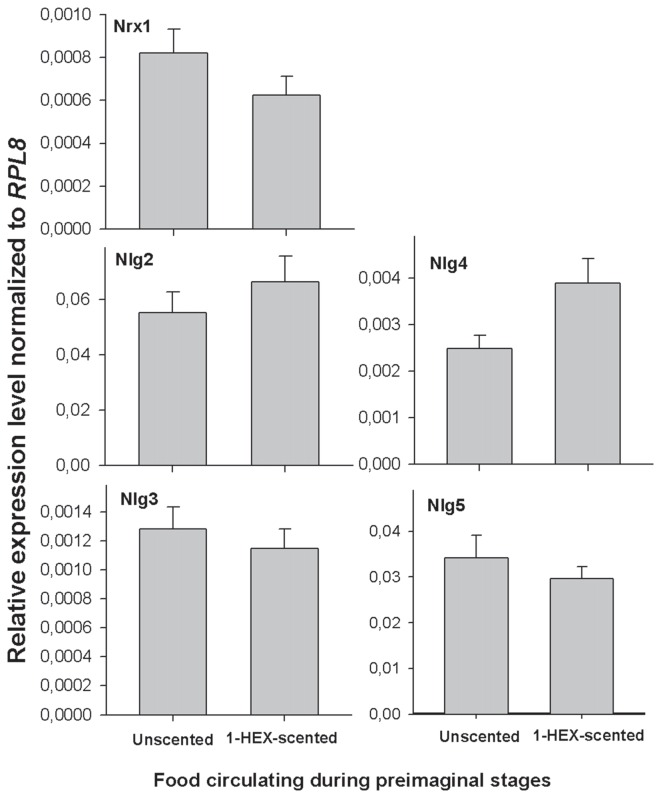
**Expression of *neuroligins (Nlgs)* and *neuroxin 1 (Nrx1)* in adult brains of animals that underwent a controlled olfactory experience at preimaginal stages.** Expression of *Nlg2–5* and *Nrx1* in adult brain tissues between honeybees reared in colonies fed 1-HEX scented sucrose solution during preimaginal stages (gray bars) or with unscented sucrose solution (black bars). Honeybee *Nlg2–5* and *Nrx1*expression were assessed by quantitative real time PCR amplification. The ribosomal gene *RPL8* was the housekeeping gene that was used as a reference level. The data are presented as the fold change in gene expression normalized to the endogenous reference gene *Rpl8* and corrected for differences in primer efficiency. *Nlg2*, neuroligin 2; *Nlg3*, neuroligin 3; *Nlg4*, neuroligin 4; *Nlg5*, neuroligin 5; *NrxI*, neurexin I.

No change was found in terms of gene expression regulation according to the preimaginal experience. The expression of gene related to synapse formation (*Nrx1, Nlg2*, *Nlg3*, *Nlg4* and *Nlg5*) showed no statistically significant among treatments (MANOVA, Wilks, F_(5,11)_ = 0, 61, *p* = 0.29; Figure 3).

### The Effect of Preimaginal Experience on EAG Recordings

**Figure 4:**
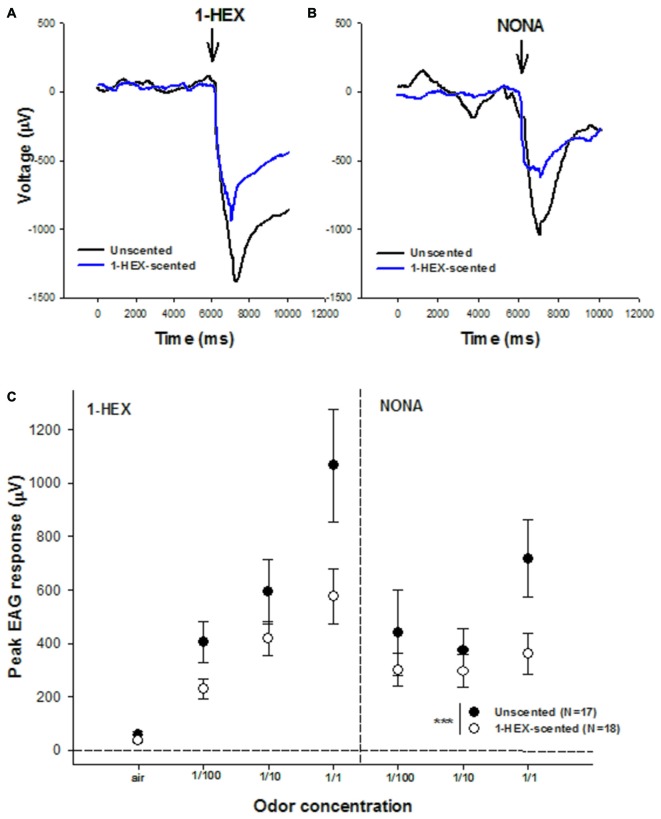
**Electrical recordings of antennae from bees of 3–5 days of age that underwent a controlled olfactory experience at preimaginal stages.** Samples of electroantennogram (EAG) recordings in response to 1-HEX **(A)** and NONA **(B)** are recorded from antennae of bees with (black line) and without (blue line) preimaginal experiences. **(C)** Mean values (± standard error) of electrical recordings of antennae of bees with (white dots) and without (black dots) pre-imaginal experiences. Three different concentrations of 1-HEX and Nonanal were tested. Asterisks indicate statistical differences in a Generalized Linear Model (GLM) (***p < 0.0005). The number of recordings is shown between brackets.

Electrophysiology records by means of EAGs were measured in antenna of 3–5 day-old bees in order to evaluate if preimaginal olfactory experiences modulate the sensitivity of olfactory receptors (Figures 4A,B). The analysis showed statistical differences between EAG records obtained in each group (GLM, *p* = 0.0003, Figure 4C). In general, regardless of the odor identity and its concentration, antennal response of bees with the olfactory pre-exposure experiences showed lower EAG records compared to the antennal response of bees reared in colonies fed unscented food during their larval stages. We found that the higher the odor concentration used to stimulate the antennae, the relatively lower the neural activity of this sensory appendage.

### The Effect of Preimaginal Experience on Sensilla Number

**Figure 5:**
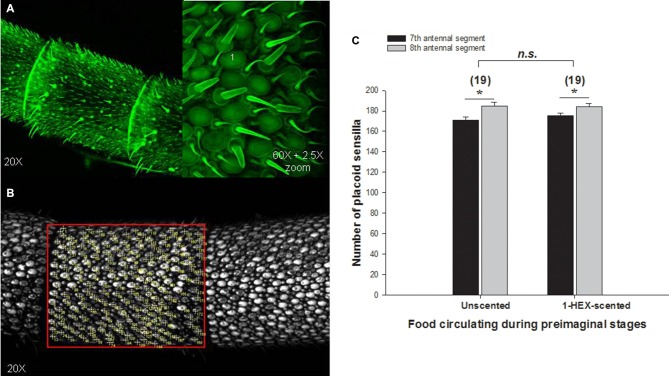
**Quantitative analysis of olfactory placoid sensilla in adults that underwent a controlled olfactory experience at preimaginal stages.** Antennae of 3–5 day-old bees were observed under confocal microscope **(A)** to count the number plate-like or placoid sensilla in the 7th and 8th antennal segments of the flagellum. The number of placoid sensilla per segment was quantified **(B)**. We compared placoid sensilla number between segments and between antennae of bees with or without the preimaginal olfactory experience **(C)**. Asterisks indicate significant differences in a two-way analysis of variance (ANOVA; *p < 0.05). The number of antennae tested is shown between brackets.

To evaluate the effect of preimaginal olfactory experiences on the antennal morphology, the number of placoid sensilla was counted in the antennae of 3–5 day-old adult bees (Figures 5A,B). The number of placoid sensilla in the antenna of young bees with or without preimaginal olfactory experiences did not differ. Significant differences were observed between 7th and 8th segments (Factor segment analyzed: F_(1,36)_ = 16.97, *p* = 0.0002; Figure 5C), however, results do not reveal significant differences neither between the experimental groups nor in the interaction between olfactory experience and antennal segments, (Olfactory experiences Factor: F_(1,36)_ = 0.726, *p* = 0.614; Interaction among factors F_(1,36)_ = 0.79, *p* = 0.379; Figure 5C).

## Discussion

Until now, little is known about the impact of larval olfactory experiences on the food related behavior of adult honeybees and the development of their olfactory system. Our results show that experiences with 1-HEX-scented food at preimaginal stages impacted both the physiology and behavior of young adult honeybees. At the behavioral level, we detected that young bees showed a higher probability to respond to the experienced odor (1-HEX) throughout the four conditioning trials. Consistently, bees with preimaginal experience responded more to 1-HEX than to any other odor. Preimaginal experience also affected similar novel odors such as HEXA. Preimaginal experiences did not induce a clear differential expression of two proteins (*Nrx* and *Nlgs*) that play roles in synaptic formation. Furthermore, the effect of the olfactory experience extended to the sensory periphery and although it did not alter the morphology of the antennae (i.e., number of placoid sensilla did not change), the antennal responsiveness did decrease in the experienced group, a phenomenon that has been recently reported in olfactory conditioned honeybees (Claudianos et al., 2014).

### Survival of Olfactory Memory after Metamorphosis

The higher level of PER values found during 1-HEX conditioning can be explained by the presence of olfactory memory established at preimaginal stages by the offering of scented food that survived through metamorphosis. Although we did not test the abilities of the honeybee larvae to learn odors from the food, olfactory conditioning at this stage is supported by numerous studies in different groups of holometabolous insects. Fruit fly* Drosophila* was successfully trained in a differential conditioning in which two odorants were paired with a positive or negative gustatory reinforcement (Scherer et al., 2003; Zeng et al., 2007). Similarly it was conditioned to visual cues (Gerber et al., 2004). Larvae of *Spodoptera littoralis* were highly attracted to odors experienced in the food, suggesting olfactory memory formation (Carlsson et al., 1999). *Manduca sexta* caterpillars learned to associate aversive stimuli with neutral odors, which led to memories that survived through two larval molts and into the adulthood (Blackiston et al., 2008). A similar long-lasting avoidance response was observed in the fly *Drosophila* (Tully et al., 1994) after aversive conditioning to an odor during the third and fifth instar of its larvae stage. In an appetitive context, preferences for pollen odors in the solitary-pollen-specialist bee *Colletes fulgiduslongi plumosus* were influenced by the diet on which bees were reared as larvae (Dobson, 1987).

We speculate that the offering of scented food (e.g., Arenas et al., 2007) enables larvae to associatively learn the odor with the reward. Inside the colony, the liquid food (nectar) is distributed rapidly among nestmates through mouth-to-mouth food exchanges (Nixon and Ribbands, 1952). During these interactions, olfactory and gustatory cues of the nectar propagate among bees of all ages (Farina et al., 2005; Grüter et al., 2006) including larvae (Crailsheim, 1998). Indeed we observed that, the food we supplied had been used in the nourishment of the larvae (“Supplementary Figure S1”). On the other hand, it is well known that some honeybee brain progenitor cells present at the larval stage are retained through metamorphosis (Farris et al., 1999), making plausible the survival of a memory trace in the adult. Capability of larvae to retain a neutral stimulus paired with a gustatory reinforcement seems to be the most plausible explanation for the behavioral plasticity we observed in the adulthood.

It is worth mentioning that there are different types of social or environmental cues that could signal variation during larval development affecting adult behaviors. In this regard, the imprinting process by which an animal acquires a permanent reference pattern from a stimulus with irreversible impact onto adult behavior (Lorenz, 1935) was suggested in the acquisition of cues that mediate nest mates recognition in the ant *Cataglyphis cursor* (Isingrini et al., 1985). However, imprinting seems not to be involved in the acquisition of sensory experiences as established in our work. Bees showed higher response towards the experienced odor compared to the control group when tested at 3–5 days of age but not later (i.e., at 17–19 days of age, Figures 1, 2, and “Supplementary Figure S2”). Hence the effect of the preimaginal experience seems to decay with time instead of leading to irreversible olfactory-driven preferences that would be expected in an imprinting-like phenomenon (Gascuel and Masson, 1987).

Barron and Corbet (1999) suggested that changes in adult response to exposed odors in *Drosophila* was not due to preimaginal conditioning, but due to “chemical legacy”, a phenomenon by which compounds carried over from the larval stage are learned at the moment of the adult emergence. We reasoned that changes we observed were less likely to be learned in a “chemical legacy” like process (Barron and Corbet, 1999). On the one hand, we paid much attention to avoid odor contamination out of the larval period, by moving the capped brood to a non-experimentally-scented environment and by controlling thoroughly the cleanness of the incubator at the time of emergence. On the other hand, we chose 1-HEX as stimulation odor since it presents a high vapor pressure (0.928 mmHg), which runs against the possibility odorant molecules to persist for long periods, even if trapped inside the capped cells.

In accordance with preimaginal olfactory learning, we observed that the PER response was specific and did not extend to novel odors except for the most similar one (Figure 2). It is not new that honeybees can generalize previously acquired cues to novel cues sharing common features (Deisig et al., 2002; Guerrieri et al., 2005). In nature, where stimuli are oddly presented in the same manner twice, generalization enables the animal to continue responding when cues are slightly different from the previously learned (Pearce, 1987). We observed for the first time generalization to an odor experienced at preimaginal stages. In our experiments, this effect was strong to HEXA but moderated for 1-NON and NONA, the odors least chemically and perceptually similar to the experienced odor (see Guerrieri et al., 2005). Consistent with the incapacity of mature bees of 17–19 day of age to recover larval experiences, lower PER probability to novel odors were detected in this group. Initial response to 1-HEX (and probably to NONA) showed the tendency to be higher than to the other odors even in the group which was fed unscented food. Although not significant, higher levels of response could be due to innate differences in the salience of these odors for honeybees, such that more salient odors would induce higher levels of response. Furthermore, we cannot rule out the contribution of alternative preimaginal experience-dependent preferences to 1-HEX, since in our experiments, bees were reared in colonies that in addition to the offering of 1-HEX scented or unscented food, were free to visit natural food sources.

How preimaginal memories are retained throughout the transition for larvae to adult is poorly understood. Olfactory memory is known to reside in the mushroom bodies and probably in the antennal lobes of the larval and adult insect brain (Davis et al., 1993; de Belle and Heisenberg, 1994; Arenas et al., 2009, 2012). Mushroom bodies undergo extensive remodeling that includes the pruning of larval neurons to the cell body before the formation of the adult-specific circuits, leading us to speculate that larval memories can be stored within a subset of neural circuits that survived through metamorphosis (Farris et al., 1999).

### Plasticity of the Adult Nervous System After Preimaginal Experience

By considering the hypothesis that memories are established in honeybee preimago, we also investigated whether *Nrx* and *Nlgs*, two proteins whose expressions have been linked to synaptic formation and associative learning in insects (Zeng et al., 2007; Biswas et al., 2008, 2010), are differently regulated on adults by larval experiences. It is already known that *Nrx-1* is required for associative learning in *Drosophila* larvae (Zeng et al., 2007). Also *Nrx* and *Nlgs* are found to be up-regulated in the honeybee adult brain after associative learning (Biswas et al., 2010). Contrarily as we expected, we found no changes in the expression of these proteins on young adult brains to the specific experience with the odor. An up-regulation of these proteins would suggest the incipient formation of neural connections at the early stages, and hence, the possibility of being regulated by external sensory inputs during development. A previous study reported that expression of *Nrx-1* and* Nlg1–5* in honeybee brains present pronounced up-regulation from pupal stages, but they maintained least expression levels during larval stages (Biswas et al., 2008). This consideration might be one of the explanations for the lack of changes in their expression after the preimaginal experience.

There is evidence that the olfactory receptors, the first point of neural contact for odorant molecules, contributed to the olfactory plasticity of adult bees. Some studies showed that EAG responses increased with odor learning (De Jong and Pham-Delègue, 1991; Wadhams et al., 1994). Others detected no effects (Bhagavan and Smith, 1997; Sandoz et al., 2001). Our results showed, that young honeybees that had received a controlled olfactory experience at preimaginal stages exhibited diminished antennal responses compared to controls. Decrease in the neural responsiveness of the antenna is consistent with findings recently reported by Claudianos et al. (2014). The authors discussed that increasing the sensory acuity of the neural circuits to detect odorants after learning might require the down-regulation of olfactory receptors, which leads to a selectively lower responsiveness of the antennae to the experienced odors whereas it enables the animal to remain receptive to new scents. They further suggest that a reduced number of receptors can still be enough to detect learned odorants, as they become very salient for the bees after learning. This evidence and our results support the idea that the peripheral sensory system is not hard-wired but plastic, modulating its response depending on rewarded experiences with odors. One important finding of our study was that the antennal responsiveness was also affected by the novel tested odor, NONA, suggesting that generalization phenomenon also take place at the PNS. Since EAG measurements quantify the response of the entire antenna, and floral scent receptors are often broadly tuned to many odorant molecules, we do not rule out that detection of both 1-HEX and NONA might occur by the same family of receptors that were modulated by the experience.

Decreases detected in EAGs should not be attributed to changes in the number of placoid sensilla of individuals that underwent the olfactory experience at the larval stage. As we showed here, they remained constant, at least in the 7th and 8th antennal segments, which were suggested to be good candidates to represent other segments of the antenna in terms of olfactory sensilla (Riveros and Gronenberg, 2010). Altogether, these results stress the complexity of the chemosensory periphery and the olfactory system as a whole and highlight the role of experiences at early stages of development on physiological and behavioral plasticity.

In honeybees, like in other holometabolous insects, adults and larvae are anatomically different and present distinct behavioral repertories (Anderson, 1972). In particular, adult honeybees progress through different tasks inside the hive until they become foragers (Seeley, 1982), which require sophisticated odor-driven behavior to find suitable food sources and to navigate back to the nest (Sigg et al., 1997). Larvae, in contrast, live in confinement inside their cells where they are fed by nurse bees (Wilson, 1971). They do not need long-range odor detection or complex behavioral repertoires to get the food. Despite these differences, larval and adult diets are based on nectar and pollen (Hanser and Rembold, 1964; Kunert and Crailsheim, 1987). In this regard, it is plausible that olfactory experiences survive metamorphosis to influence food choices of adults. Even when we failed to show retrieval of larval memories at foraging ages (17–19 days of age), biases for odors in young adults can still influence foraging decision at the colony level. For example, it is known that food processors showed more interactions with foragers carrying the experienced odor than a novel odor. With an increasing number of trophallaxes, bees with larval experiences could speed up the unloading of familiar-scented nectars (Goyret and Farina, 2005) and motivate foragers to continue visiting the source (but also recruiting more bees to it). On the other hand, neglected foragers would become less motivated to keep on active on their food sources. Similarly, experienced bees were successfully recruited to a feeding site characterized with the odor they learned inside the hive 8 days earlier (Balbuena et al., 2012). Beside the odor-specific effect on later behavioral responses we detected here, the olfactory stimulation *per se* might also contribute to the ontogeny and development of the olfactory system. This for sure would improve cognitive and perceptual capabilities on the adults affecting their individual performance and the display of social behaviors.

### Concluding Remarks

Our study provides new insight into the effects of preimaginal experiences in the honeybee and the mechanisms underlying olfactory plasticity in holometabolous insects. We found evidence that larval experience on the appetitive context induced transient changes in the behavior of adults. Whether the preimaginal experience led to an associative learning or a non-associative effect is under debate. We think that there are different pieces of information that suggest that an associative learning process is involved. The first one is the high probability to respond to 1-HEX at young ages and the decay of the response later in life. The second issue is the increased probability to respond to a novel odor similar to the experienced one. Memory decay and generalization are both consistent with an associative learning process. Behavioral changes detected in 3–5 day-old bees were accompanied at least by changes in odor detection in the sensory periphery. We believe our results support the idea that larval experience has subsequent effects on odor processing and perception, contributing to the neuronal plasticity of the olfactory system.

## Author Contributions

Conceived and designed the experiments: GR, AA, WMF. Performed the experiments: GR, CF. Analyzed the data: GR, CF, JPG. Contributed reagents/materials/analysis tools: PA, WMF. Wrote the article: GR, AA, WMF.

## Funding

Funding was provided by the Consejo Nacional de Investigaciones Científicas y Técnicas (CONICET), the Agencia Nacional de Promoción Científica y Tecnológica (ANPCYT) and the University of Buenos Aires.

## Conflict of Interest Statement

The authors declare that the research was conducted in the absence of any commercial or financial relationships that could be construed as a potential conflict of interest.
