# Supplementary material for: Odor Experiences during Preimaginal Stages Cause Behavioral and Neural Plasticity in Adult Honeybees
Source: Front Behav Neurosci. 2016 Jun 3;10:105. doi: 10.3389/fnbeh.2016.00105 (PMC4891344; doi:10.3389/fnbeh.2016.00105)
Supplement: Supplementary file 1 [file Presentation_1.pdf]

Supplementary Material

Odor experiences during preimaginal stages cause behavioral and neural plasticity in adult honeybees

Gabriela Ramírez, Carol Fagundez, Juan P. Grosso, Pablo Argibay, Andrés Arenas and Walter M. Farina[*¶]

***Corresponding author**: e-mail: walter@fbmc.fcen.uba.ar*

Materials and Methods

PER to 1-HEX and to novel odors

Testing odors experienced at the adult stage

In order to control for the formation of olfactory memories by means of the offering of scented food, adult workers from stimulation hives were captured and tested to the odor presentation in the PER paradigm (Takeda 1961, Bitterman et al. 1983). The offering of food (scented and unscented) into the stimulation colonies was carried out as detailed in "***Study site, colonies and caged-bees".*** Food was colored with a red dye (red amaranth, Saporiti ®) to determinate if the food that was offered into a 1.5L in-hive feeder was successfully transferred to the storages of the nest (***Figure 1S***). In hive workers of unknown ages were captured directly at the entrance of the hives, 1 day after the in-hive feeders were refilled with either scented or unscented food as appropriate (i.e. the food was circulating within the nest). Afterwards the bees were prepared as we explained in "***Testing behavioral responses"*** (i.e. harnessed and kept in an incubator until being tested). Memory retention to the stimulation odor (1-HEX) and generalization to three novel odors (1-NON, NONA and HEXA) were evaluated following the procedure explained in "***PER to 1-HEX and to novel odors***".

Testing preimaginal experiences in bees reared within the hive

With the aim to determine if the heterogeneity of the rearing environment affected the PER of mature control bees and mature bees that underwent a preimaginal experience (see "***Odor experience during the larval stage***"), experimental bees were confined into wooden cages that were exposed to a beehive environment. Bee cages were placed inside host colonies since their 5 days of age and remained inside the hive until the testing day, at 17-19 days of age. The host colonies were commercial hives of our apiary containing about 40.000 European honeybees, which were never used in experiments. They provided a more natural environment to the caged bees than the incubator (see "***Study site, colonies and caged-bees***") including physical, visual and chemical cues that were probably absent when reared under laboratory conditions. Bee cages were located on one side of the hive and between two crowded frames of brood, honey and pollen. Although the experimental bees had feeders inside the cages with unscented sugar solution, antennation and food exchange with colony bees was allowed through thelateral screen of the boxes. At the age of 17-19 days of age bees were prepared for testing as explained in "***Testing behavioral responses*".** Memory retention to the stimulation odor (1-HEX) and generalization to three novel odors (1-NON, NONA and HEXA) were evaluated following the procedure explained in "***PER to 1-HEX and to novel odors***".

Results

Testing odors experienced at the adult stage

Here we evaluated if the offering of scented food enabled the formation of olfactory memories in adult in-hive workers. For hive bees, the ANOVA-RM showed statistical

difference in the interaction between tested odors and odor experiences ($F_{3,348}$ =15.363, $p < 0.0001$; RM-ANOVA, ***Figure 2S A***). Posterior simple effects revealed that bees from hive fed 1-HEX-scented food showed higher PER values to all the four odors tested compared to those bees stimulated with unscented food (1-HEX:$F_{1,464}$ = 92.892, $p < 0.0001$, HEXA: $F_{1,464}$= 11.379, p = 0.0008 and NONA: $F_{1,464}$ = 8.360, p =0.004, ***Figure 2S A***).

Moreover, simple-effect analysis also revealed statistical differences among odors within the group of bees that had been reared in the colony fed 1-HEX (Simple-effect ANOVA $F_{3,464}$ = 33.083, $p < 0.0001$; ***Figure 2S A***). *Post hoc* comparisons for this colony showed statistical differences between PER levels to 1-HEX compared to HEXA, 1-NON and NONA ($p < 0.05$; Tukey comparisons).

Testing preimaginal experiences in bees reared within the hive

Here we analyzed if the rearing environment had any influence into the response to the odors. Seventeen-19-day old bees showed no statistical differences between treatments (i.e. bees fed unscented food and bees fed 1-HEX scented-food at their larval stage) in a two-way RM-ANOVA ($F_{3, 201}$ = 1.233, p = 0.298, ***Figure 2S B***).

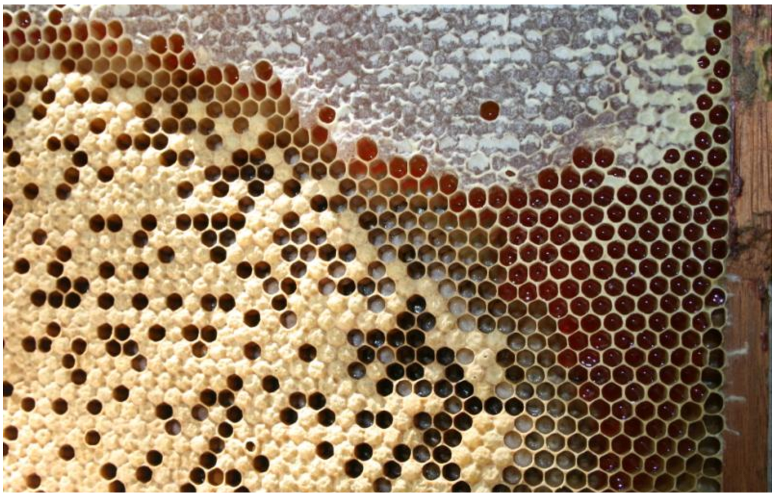

Figure 1S: Colored food in the storing area of a honeybee comb. As we showed in the picture, the red dyed contained in the food was storage near the brood, where it is likely to be used as food for the larvae.

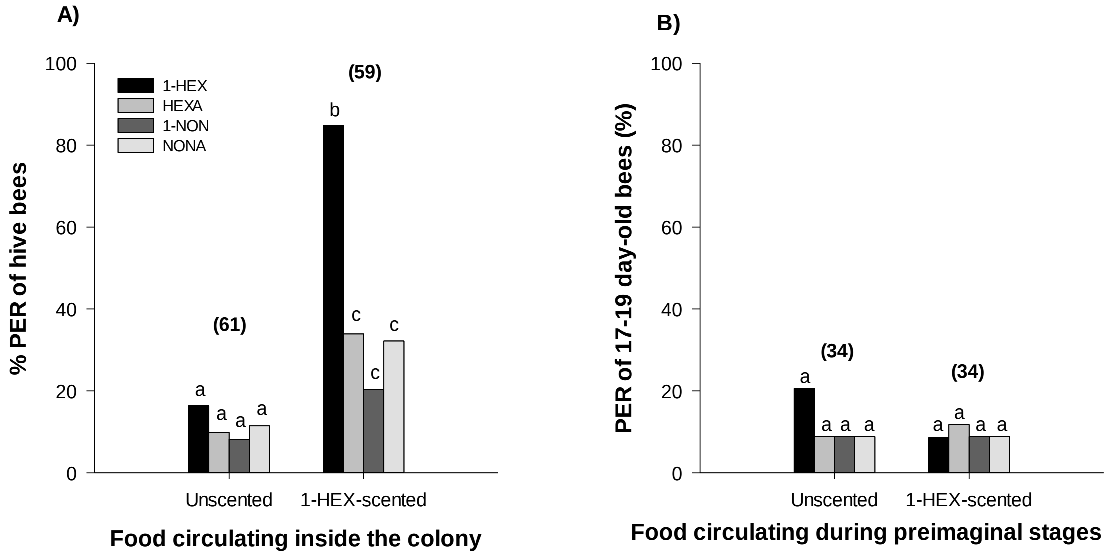

Figure 2S: Response to the stimulation odor and to novel odors in adults that underwent controlled olfactory experiences inside the colony. Proboscis extension responses (PER) of unknown age bees (**A**) and 17/19-day-old bees (**B**) to the odor experienced into the hive (1-hexanol, 1-HEX) and to three novel odors (hexanal, HEXA; 1-nonanol, 1-NON; and nonanal, NONA). The bees were captured in stimulation hives where circulated either unscented food or 1-HEX-scented food. In B) tested bees belonged to a hive fed 1-HEX-scented food during their preimaginal lifespan; then they were reared in the laboratory until 5 days after emergence and finally reintroduced in their colony till testing at 17-19 days of age. Different letters indicate significant differences in a two-way RM-ANOVA. The number of tested bees is shown in brackets.
